# Supplementary figures and images for: Artificial light at night correlates with seabird groundings: mapping city lights near a seabird breeding hotspot
Source: PeerJ. 2022 Oct 18;10:e14237. doi: 10.7717/peerj.14237 (PMC9586080; doi:10.7717/peerj.14237)

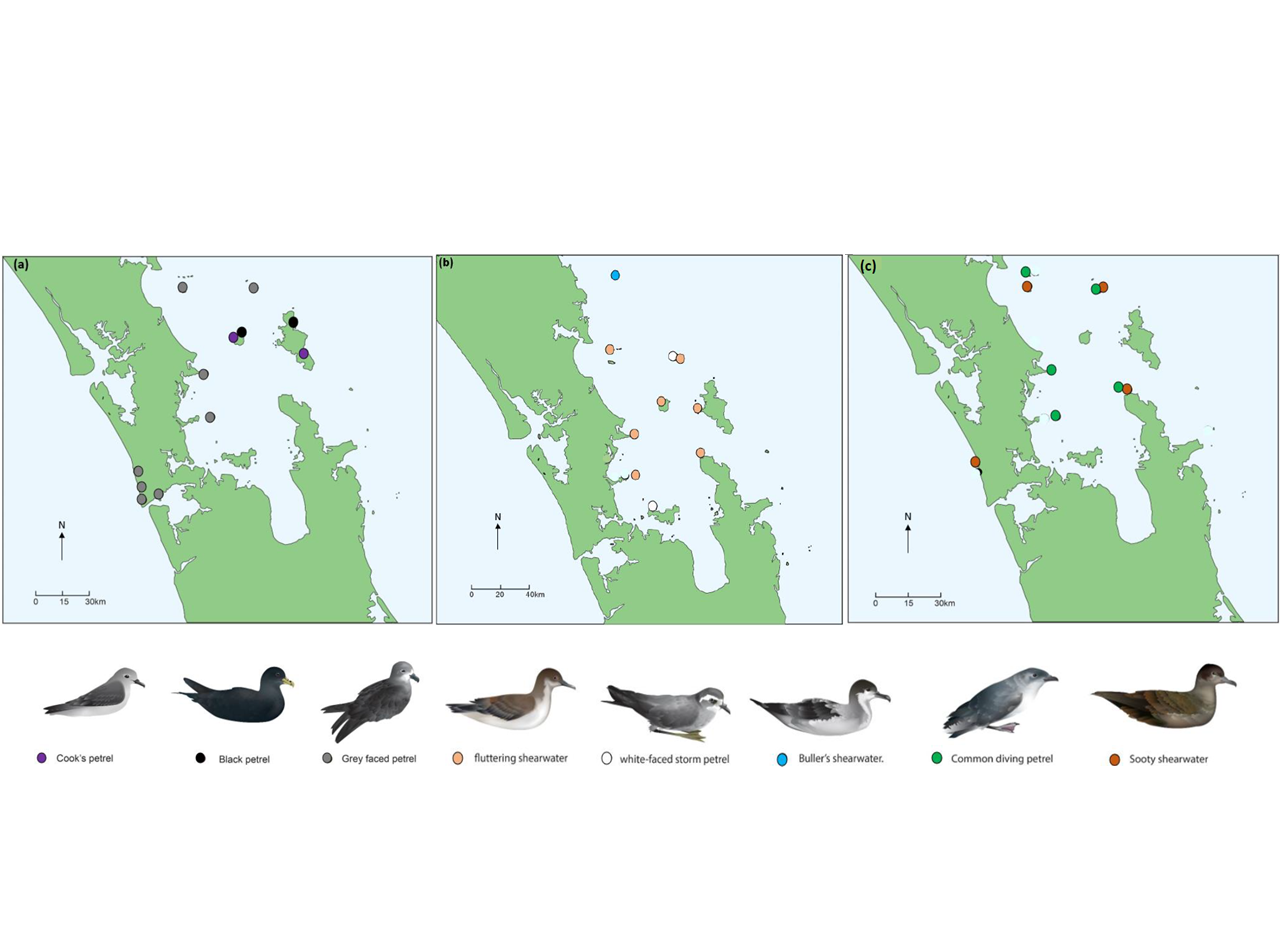

Supplement: Supplemental Information 1 [file peerj-10-14237-s001.png]
