# Supplementary material for: Artificial light at night correlates with seabird groundings: mapping city lights near a seabird breeding hotspot
Source: PeerJ. 2022 Oct 18;10:e14237. doi: 10.7717/peerj.14237 (PMC9586080; doi:10.7717/peerj.14237)
Supplement: Supplemental Information 2 [file peerj-10-14237-s002.pdf]

Online supplementary Table 1: Output of the pairwise contrasts between different seabird species and the location (rural vs urban). * and bold indicated significance. Pseudo R2 is 0.08.

| **contrast** | **t ratio** | **p value** |
|---|---|---|
| Black petrel (rural) - Buller's shearwater (rural) | 6.704 | **0.0001*** |
| Black petrel (rural) - Common diving petrel (rural) | 5.651 | **0.001*** |
| Black petrel (rural) - Cook's petrel (rural) | -2.144 | 0.7216 |
| Black petrel (rural) - Fluttering shearwater (rural) | 4.604 | **0.0104*** |
| Black petrel (rural) - Grey-faced petrel (rural) | -1.517 | 0.9675 |
| Black petrel (rural) - Sooty shearwater (rural) | 5.118 | **0.0034*** |
| Black petrel (rural) - White-faced storm petrel (rural) | 5.701 | **0.0009*** |
| Black petrel (rural) - Black petrel (urban) | 6.915 | **0.0001*** |
| Black petrel (rural) - Buller's shearwater (urban) | 6.655 | **0.0001*** |
| Black petrel (rural) - Common diving petrel (urban) | 6.121 | **0.0004*** |
| Black petrel (rural) - Cook's petrel (urban) | -11.603 | **<.0001*** |
| Black petrel (rural) - Fluttering shearwater (urban) | 5.316 | **0.0022*** |
| Black petrel (rural) - Grey-faced petrel (urban) | 3.829 | **0.0534*** |
| Black petrel (rural) - Sooty shearwater (urban) | 5.332 | **0.0021*** |
| Black petrel (rural) - White-faced storm petrel (urban) | 5.986 | **0.0005*** |
| Buller's shearwater (rural) - Common diving petrel (rural) | -2.251 | 0.6579 |
| Buller's shearwater (rural) - Cook's petrel (rural) | -11.243 | **<.0001*** |
| Buller's shearwater (rural) - Fluttering shearwater (rural) | -4.043 | **0.0344*** |
| Buller's shearwater (rural) - Grey-faced petrel (rural) | -7.94 | **<.0001*** |
| Buller's shearwater (rural) - Sooty shearwater (rural) | -3.686 | **0.0711*** |
| Buller's shearwater (rural) - White-faced storm petrel (rural) | -2.467 | 0.5246 |
| Buller's shearwater (rural) - Black petrel (urban) | -5.732 | **0.0009*** |
| Buller's shearwater (rural) - Buller's shearwater (urban) | -0.963 | 0.9996 |
| Buller's shearwater (rural) - Common diving petrel (urban) | -2.243 | 0.6625 |
| Buller's shearwater (rural) - Cook's petrel (urban) | -31.826 | **<.0001*** |
| Buller's shearwater (rural) - Fluttering shearwater (urban) | -4.022 | **0.0359*** |
| Buller's shearwater (rural) - Grey-faced petrel (urban) | -5.885 | **0.0006*** |
| Buller's shearwater (rural) - Sooty shearwater (urban) | -3.718 | 0.0666 |

| contrast | t ratio | p value |
|---|---|---|
| Buller's shearwater (rural) - White-faced storm petrel (urban) | -2.474 | 0.5203 |
| Common diving petrel (rural) - Cook's petrel (rural) | -9.916 | **<.0001*** |
| Common diving petrel (rural) - Fluttering shearwater (rural) | -1.833 | 0.8782 |
| Common diving petrel (rural) - Grey-faced petrel (rural) | -6.95 | **0.0001*** |
| Common diving petrel (rural) - Sooty shearwater (rural) | -1.156 | 0.9972 |
| Common diving petrel (rural) - White-faced storm petrel (rural) | 0.002 | 1 |
| Common diving petrel (rural) - Black petrel (urban) | -3.041 | 0.2318 |
| Common diving petrel (rural) - Buller's shearwater (urban) | 2.076 | 0.7606 |
| Common diving petrel (rural) - Common diving petrel (urban) | 2.175 | 0.7035 |
| Common diving petrel (rural) - Cook's petrel (urban) | -27.487 | **<.0001*** |
| Common diving petrel (rural) - Fluttering shearwater (urban) | -1.08 | 0.9986 |
| Common diving petrel (rural) - Grey-faced petrel (urban) | -3.069 | 0.2212 |
| Common diving petrel (rural) - Sooty shearwater (urban) | -0.982 | 0.9995 |
| Common diving petrel (rural) - White-faced storm petrel (urban) | 0.397 | 1 |
| Cook's petrel (rural) - Fluttering shearwater (rural) | 8.692 | **<.0001*** |
| Cook's petrel (rural) - Grey-faced petrel (rural) | 0.157 | 1 |
| Cook's petrel (rural) - Sooty shearwater (rural) | 9.445 | **<.0001*** |
| Cook's petrel (rural) - White-faced storm petrel (rural) | 10.095 | **<.0001*** |
| Cook's petrel (rural) - Black petrel (urban) | 6.724 | **0.0001*** |
| Cook's petrel (rural) - Buller's shearwater (urban) | 11.167 | **<.0001*** |
| Cook's petrel (rural) - Common diving petrel (urban) | 10.252 | **<.0001*** |
| Cook's petrel (rural) - Cook's petrel (urban) | -38.835 | **<.0001*** |
| Cook's petrel (rural) - Fluttering shearwater (urban) | 8.961 | **<.0001*** |
| Cook's petrel (rural) - Grey-faced petrel (urban) | 6.471 | **0.0002*** |
| Cook's petrel (rural) - Sooty shearwater (urban) | 9.044 | **<.0001*** |
| Cook's petrel (rural) - White-faced storm petrel (urban) | 10.083 | **<.0001*** |
| Fluttering shearwater (rural) - Grey-faced petrel (rural) | -5.957 | **0.0005*** |
| Fluttering shearwater (rural) - Sooty shearwater (rural) | 0.794 | 1 |
| Fluttering shearwater (rural) - White-faced storm petrel (rural) | 1.897 | 0.8509 |
| Fluttering shearwater (rural) - Black petrel (urban) | -1.093 | 0.9984 |
| Fluttering shearwater (rural) - Buller's shearwater (urban) | 3.907 | **0.0455*** |

| Contrast | t ratio | p value |
|---|---|---|
| Fluttering shearwater (rural) - Common diving petrel (urban) | 2.647 | 0.4187 |
| Fluttering shearwater (rural) - Cook's petrel (urban) | -24.361 | **<.0001*** |
| Fluttering shearwater (rural) - Fluttering shearwater (urban) | 3.337 | 0.1384 |
| Fluttering shearwater (rural) - Grey-faced petrel (urban) | -1.103 | 0.9983 |
| Fluttering shearwater (rural) - Sooty shearwater (urban) | 1.005 | 0.9994 |
| Fluttering shearwater (rural) - White-faced storm petrel (urban) | 2.343 | 0.601 |
| Grey-faced petrel (rural) - Sooty shearwater (rural) | 6.44 | **0.0002*** |
| Grey-faced petrel (rural) - White-faced storm petrel (rural) | 6.997 | **0.0001*** |
| Grey-faced petrel (rural) - Black petrel (urban) | 5.476 | **0.0015*** |
| Grey-faced petrel (rural) - Buller's shearwater (urban) | 7.898 | **<.0001*** |
| Grey-faced petrel (rural) - Common diving petrel (urban) | 7.441 | **<.0001*** |
| Grey-faced petrel (rural) - Cook's petrel (urban) | -8.286 | **<.0001*** |
| Grey-faced petrel (rural) - Fluttering shearwater (urban) | 6.747 | **0.0001*** |
| Grey-faced petrel (rural) - Grey-faced petrel (urban) | 8.643 | **<.0001*** |
| Grey-faced petrel (rural) - Sooty shearwater (urban) | 6.764 | **0.0001*** |
| Grey-faced petrel (rural) - White-faced storm petrel (urban) | 7.329 | **<.0001*** |
| Sooty shearwater (rural) - White-faced storm petrel (rural) | 1.209 | 0.9957 |
| Sooty shearwater (rural) - Black petrel (urban) | -1.943 | 0.8297 |
| Sooty shearwater (rural) - Buller's shearwater (urban) | 3.514 | 0.0994 |
| Sooty shearwater (rural) - Common diving petrel (urban) | 2.024 | 0.7887 |
| Sooty shearwater (rural) - Cook's petrel (urban) | -25.895 | **<.0001*** |
| Sooty shearwater (rural) - Fluttering shearwater (urban) | 0.145 | 1 |
| Sooty shearwater (rural) - Grey-faced petrel (urban) | -1.948 | 0.827 |
| Sooty shearwater (rural) - Sooty shearwater (urban) | 1.277 | 0.9927 |
| Sooty shearwater (rural) - White-faced storm petrel (urban) | 1.683 | 0.93 |
| White-faced storm petrel (rural) - Black petrel (urban) | -3.123 | 0.2019 |
| White-faced storm petrel (rural) - Buller's shearwater (urban) | 2.269 | 0.6468 |
| White-faced storm petrel (rural) - Common diving petrel (urban) | 0.754 | 1 |
| White-faced storm petrel (rural) - Cook's petrel (urban) | -27.848 | **<.0001*** |
| White-faced storm petrel (rural) - Fluttering shearwater (urban) | -1.129 | 0.9978 |
| White-faced storm petrel (rural) - Grey-faced petrel (urban) | -3.152 | 0.1922 |

| **Contrast** | **t ratio** | **p value** |
|---|---|---|
| White-faced storm petrel (rural) - Sooty shearwater (urban) | -1.026 | 0.9992 |
| White-faced storm petrel (rural) - White-faced storm petrel (urban) | 2.094 | 0.7507 |
| Black petrel (urban) - Buller's shearwater (urban) | 5.579 | **0.0012*** |
| Black petrel (urban)- Common diving petrel (urban) | 4.168 | **0.0264*** |
| Black petrel (urban) - Cook's petrel (urban) | -21.016 | <.0001 |
| Black petrel (urban) - Fluttering shearwater (urban) | 2.319 | 0.616 |
| Black petrel (urban) - Grey-faced petrel (urban) | -0.033 | 1 |
| Black petrel (urban) - Sooty shearwater (urban) | 2.324 | 0.6127 |
| Black petrel (urban) - White-faced storm petrel (urban) | 3.803 | 0.0563 |
| Buller's shearwater (urban) - Common diving petrel (urban) | -1.944 | 0.8292 |
| Buller's shearwater (urban) - Cook's petrel (urban) | -31.558 | **<.0001*** |
| Buller's shearwater (urban) - Fluttering shearwater (urban) | -3.816 | 0.0548 |
| Buller's shearwater (urban) - Grey-faced petrel (urban) | -5.725 | **0.0009*** |
| Buller's shearwater (urban) - Sooty shearwater (urban) | -3.525 | 0.0974 |
| Buller's shearwater (urban) - White-faced storm petrel (urban) | -2.209 | 0.6831 |
| Common diving petrel (urban) - Cook's petrel (urban) | -28.706 | **<.0001*** |
| Common diving petrel (urban) - Fluttering shearwater (urban) | -2.078 | 0.7597 |
| Common diving petrel (urban) - Grey-faced petrel (urban) | -4.277 | **0.021*** |
| Common diving petrel (urban) - Sooty shearwater (urban) | -1.912 | 0.844 |
| Common diving petrel (urban) - White-faced storm petrel (urban) | -0.372 | 1 |
| Cook's petrel (urban) - Fluttering shearwater (urban) | 25.228 | **<.0001*** |
| Cook's petrel (urban) - Grey-faced petrel (urban) | 21.178 | **<.0001*** |
| Cook's petrel (urban) - Sooty shearwater (urban) | 25.012 | **<.0001*** |
| Cook's petrel (urban) - White-faced storm petrel (urban) | 28.025 | **<.0001*** |
| Fluttering shearwater (urban) - Grey-faced petrel (urban) | -2.381 | 0.5775 |
| Fluttering shearwater (urban) - Sooty shearwater (urban) | 0.079 | 1 |
| Fluttering shearwater (urban) - White-faced storm petrel (urban) | 1.682 | 0.9301 |
| Grey-faced petrel (urban) - Sooty shearwater (urban) | 2.385 | 0.575 |
| Grey-faced petrel (urban) - White-faced storm petrel (urban) | 3.903 | 0.0459 |
| Sooty shearwater (urban) - White-faced storm petrel (urban) | 1.541 | 0.9632 |
